# Reciprocal functional interactions between the brainstem and the lower spinal cord

**DOI:** 10.3389/fnins.2014.00124

**Published:** 2014-05-30

**Authors:** Itaru Yazawa

**Affiliations:** Laboratory of Neural Control, National Institute of Neurological Disorders and Stroke, National Institutes of Health, Bethesda, MD, USA

**Keywords:** entrainment, hyperoxic/normocapnic state, locomotion, locomotor pattern generator, respiration

## Abstract

The interplay of the neuronal discharge patterns regarding respiration and locomotion was investigated using electrophysiological techniques in a decerebrate and arterially perfused *in situ* mouse preparation. The phrenic, tibial, and/or peroneal nerve discharge became clearly organized into discharge episodes of increasing frequency and duration, punctuated by periods of quiescence as the perfusion flow rate increased at room temperature. The modulated sympathetic tone induced by the hyperoxic/normocapnic state was found to activate the locomotor pattern generator (LPG) via descending pathways and generate a left and right alternating discharge during discharge episodes in the motor nerves. The rhythm coupling of respiration and locomotion occurred at a 1:1 frequency ratio. Although the phrenic discharge synchronized with the tibial discharge at all flow rates tested, the time lag between peaks of the two discharges during locomotion was ≈400 ms rather than ≈200 ms, suggesting spinal feedback via ascending pathways. The incidence of the phrenic and tibial discharge episodes decreased by ≈50% after spinalization at the twelfth thoracic cord and the respiratory rhythm was more regular. These results indicate that: (i) locomotion can be generated in a hyperoxic/normocapnic state induced by specific respiratory conditions, (ii) the central mechanism regarding entrainment of respiratory and locomotor rhythms relies on spinal feedback via ascending pathways, initiated by the activated LPG generating locomotion, and (iii) the increase in respiratory rate seen during locomotion is caused not only by afferent mechanical and nociceptive inputs but also by impulses from the activated spinal cord producing a locomotor-like discharge via ascending pathways.

## Introduction

Entrainment of respiratory and locomotor rhythms during locomotion is well-documented in many species, including rats, cats, rabbits, dogs, and humans (Krogh and Lindhard, 1913; Dejours, 1967; Iscoe, 1981; Bramble and Carrier, 1983; Ainsworth et al., 1996). Such coupling can be evoked by electrical stimulation of either the mesencephalic locomotor region or the subthalamic locomotor region (Bramble and Carrier, 1983; Corio et al., 1993) within a wide range of frequency ratios between locomotion and respiration (e.g., 1:1, 2:1, and 3:2). Although the coordination of respiratory and locomotor rhythms is thought to be generated by central feed forward (Eldridge et al., 1981; Krogh and Lindhard, 1913) and spinal feedback mechanisms (Brice et al., 1988; Brown et al., 1990; Potts et al., 2005; Giraudin et al., 2012), the neural mechanisms involved are poorly understood.

Pickering and Paton (2006) pioneered the use of a decerebrate and artificially perfused rat preparation to investigate brainstem function involved in respiration, circulation, and sympathetic tone, using artificial cardiopulmonary bypass to deliver oxygen to the body. Oxygen consumption goes up with increasing perfusion flow volume, as described in human extracorporeal circulation (Fox et al., 1982; Kirklin and Barratt-Boyes, 1993) presumably metabolism increases as flow rate increases. However, the increase in metabolism is not always reflected in that of neuronal activity and/or the sympathetic tone, it is unclear whether the sympathetic tone resulting from the increase in flow rate can generate unknown autonomic functions.

The present study used a decerebrate and arterially perfused *in situ* mouse preparation and electrophysiological techniques to investigate (1) whether locomotion can be autonomously generated by a certain sympathetic tone resulting from an increase in flow rate, (2) whether the rhythm coupling of locomotion and respiration can be produced during locomotion. Sequential experiments were performed progressively eliminating influencing (confounding) factors, in an attempt to unravel the central neural mechanisms that mediate the coordination of the respiratory and locomotor rhythms. Neuronal discharges in the phrenic and peroneal and/or tibial nerves were measured, which are considered to reflect the outputs that are derived in the brainstem respiratory center (Barman and Gebber, 1976) and in the spinal networks constituting the locomotor pattern generator (LPG) of the lower spinal cord. Heartbeat and systemic pressure were measured, which are considered to reflect the outputs that are derived in the cardiovascular center of the brainstem (Pickering and Paton, 2006).

## Materials and methods

Mice were kept in a temperature-controlled room with *ad libitum* access to food and water. All procedures followed a protocol approved by the National Institute of Neurological Disorders and Stroke/National Institutes of Health Animal Care and Use Committee.

### Decerebrate and arterially perfused *in situ* mouse preparation

Experiments were performed on 60 male Swiss Webster mice (Taconic Laboratory) aged 5–42 days and weighing 4.6–32.1 g. After initial sedation via inhalation of 5.0% halothane, an anesthetic combination of ketamine and xylazine (0.5–1.0 μ L/g; ketamine:xylazine 7:1) was administered intraperitoneally. During surgery, the halothane concentration was maintained at 1.5–2.0%, and the depth of anesthesia was assessed by respiratory rate and responsiveness to tail pinch. Mice were placed supine in a dissection chamber and a median laparotomy performed, from the xiphoid to the lower abdomen, for ligation and removal of the stomach, small and large intestines, spleen, pancreas, and their dominant vessels. Using a median sternotomy to open the thorax with a spreading retractor, thoracotomy was performed to allow direct visualization of the heart and lungs. After administration of heparin (10 U/L, intracardiac injection), both the pleura and the pericardium were removed, and the preparation was immediately submerged in Ringer's solution infused with a 95% O_2_–5% CO_2_ gas mixture and maintained at 5–10°C to induce suspended animation. The Ringer's solution (composition in mM: 125 NaCl, 3 KCl, 24 NaHCO_3_, 1.25 KH_2_PO_4_, 1.25 MgSO_4_, 2.5 CaCl_2_, and 10 D-glucose) was maintained at pH 7.40–7.45 after carbogenation at room temperature (Chizh et al., 1997). After cardiac arrest, craniotomy was performed, with decerebration performed with suction at the precollicular level. Mice were skinned to avoid body fluids accumulating in the subcutaneous tissues. The lungs were then removed and an incision made through the apex of the left ventricle.

**Figure 1:**
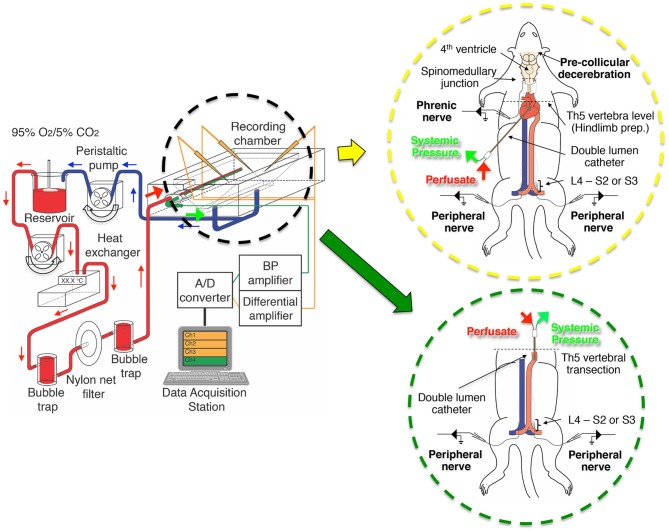
**The setup of the perfusion circuit used for electrophysiological recordings from the decerebrate and arterially perfused *in situ* mouse preparation**. The perfusate was circulated from the reservoir to the perfusion circuit with a peristaltic pump, transfused into the aortic arch of the preparation through bubble traps and net filters (nylon net pore size, 20 μm), and then recycled from the recording chamber back to the reservoir. The inset in the yellow circle is a schematic of a decerebrate and arterially perfused *in situ* mouse preparation in which a double-lumen catheter was inserted into the aortic arch via the left ventricle for perfusion of Ringer's solution, a procedure modified from that of Pickering and Paton (2006). The inset in the green circle is a schematic of the hindlimb preparation obtained by transecting a decerebrate and arterially perfused *in situ* preparation at the fifth thoracic vertebra and inserting a double-lumen catheter (NCV25GW-200W, CMP Inc., Tokyo, Japan) into the descending aorta from the severed end of the thoracic aorta for perfusion of Ringer's solution. Note that the liver was usually left *in situ* but is not shown for clarity.

While the mouse was held supine in the recording chamber, a double-lumen catheter (Φ 1.0 mm, DL-AS-040; Braintree Scientific, MA, USA) was inserted into the heart through the incision in the left ventricle (Figure 1, yellow circle). To ensure that the catheter was securely held in the aortic valve, the outer diameter of the tip was modified to a diameter slightly larger than that of the inner diameter of the value. This modification ensured the perfusate entered the ascending aorta without regurgitation into the left ventricle. Arterial perfusion was immediately initiated with carbogen-gassed, heparinized (10–20 U/L) Ringer's solution containing: Ficoll-70 (1.25–1.28%) an oncotic agent and penicillin-streptomycin-neomycin (50 U/L) to prevent infection, at room temperature. Next, an incision was made in the right atrium to maintain the internal pressure of the heart at atmospheric pressure and the incised part of the left ventricle was then sutured ensuring the catheter remained in the ascending aorta.

After resumption of spontaneous breathing at ≤15 min of the initiation of perfusion, the muscle relaxant *d*-tubocurarine (2 μ M) was added to the perfusate to induce immobilization. The left phrenic nerve was identified and detached from both blood vessels and connective tissues, and severed at the distal end. The left and right peroneal and tibial nerves were then also carefully detached at the knee level and severed at their distal ends. Although bradycardia was pronounced at the initiation of perfusion, ventricular fibrillation never developed.

Using a peristaltic pump (model 323U pump, model 318MC pump head; Watson-Marlow, Wilmington, MA, USA), the perfusate was pumped from a reservoir flask through two bubble traps and a nylon net filter (pore size, 20 μm; Figure 1). The flow rate was set to >5× the total blood volume (TBV) per minute at room temperature, with TBV calculated as 1/13 of the body weight in grams (Mitruka and Rawnsley, 1981; Harkness and Wagner, 1989). Systemic blood pressure was continuously monitored via the second lumen using a strain-gauge pressure transducer (Pressure Monitor BP-1, WPI, FL, USA). All chemicals were purchased from Sigma (St. Louis, MO, USA).

### Hindlimb preparation

For the hindlimb preparation, 12 male Swiss Webster mice (Taconic Laboratory; aged 14–31 days; weighing 8.5–30.8 g) were used. Decerebrate and arterially perfused *in situ* preparations (as above) were transected at the fifth thoracic vertebra (Figure 1, green circle). Then, the heart, lungs, and pulmonary artery and vein were removed. For perfusion, a double-lumen catheter (NCV25GW-200W; CMP Inc., Tokyo, Japan) was inserted into the descending aorta through the severed end of the thoracic aorta. After tying the thoracic aorta at the level of the sixth thoracic vertebra to prevent perfusate leakage, arterial perfusion at 5× TBV/min at room temperature was begun. The preparation began to show spontaneous left/right alternating and synchronous movements ≤10 min of perfusion initiation, *d*-tubocurarine (1–2 μ M) was administered and the left and right peroneal or tibial nerves were detached as described above.

### Extracellular recordings

Suction electrodes constructed of polyethylene tubing (PE 50; Becton, Dickinson and Company, Franklin Lakes, NJ, USA) were used to record neuronal discharge from the left phrenic and the left and right tibial or peroneal nerves. The phrenic nerve suction electrode simultaneously recorded the electrocardiogram (ECG). Phrenic nerve discharge output is considered to be derived from the brainstem respiratory center (Barman and Gebber, 1976). While, changes in heart rate and systemic pressure are indicators of changes of sympathetic tone in the cardiovascular center of the brainstem (Pickering and Paton, 2006). The peroneal and tibial nerve discharges are indicators of the outputs that are generated in the spinal network between the fourth lumbar and third sacral spinal segments and between the fourth lumbar and second sacral spinal segments, respectively. The resultant neurograms were amplified ×1000, filtered at 1–3000 Hz, and digitized using a Digidata 1320A and a Clampex (Axon Instruments, Union City, CA, USA) at sampling rates of 10,000 Hz. All data were saved for further analysis.

### Data analysis

Spinal Core software (Mor and Lev-Tov, 2007) was used to characterize/quantify the LPG output of the spinal cord induced by signal transmission from the brainstem via descending pathways and determine the correlations between the left phrenic and left peripheral muscle nerve discharges and between the left and right muscle nerve discharges during episodes of phrenic nerve discharge. Recorded data were high-pass filtered at 10–20 Hz, rectified, and compressed to a sample rate of 20 Hz. The time series data of left and right tibial nerve discharge and left phrenic and left tibial nerve discharge were used to compute the time domain between the lags from −1 to 1 s and from −2.5 to 2.5 s, respectively, and develop the correlation curves.

Statistical values are expressed as the mean (SE). Differences between means were analyzed using either a statistical software package (Sigma Stat for Windows; SPSS, Chicago, IL, USA) or excel and assessed by either one factor repeated measures ANOVA with a Scheffe *post-hoc* or the paired *t*-test. A *P* < 0.05 was considered to indicate statistical significance.

## Results

### Dependence of systemic pressure and phrenic and peripheral nerve discharge on perfusion flow rate

Although oxygen consumption goes up with increasing perfusion flow volume, as described in human extracorporeal circulation (Fox et al., 1982; Kirklin and Barratt-Boyes, 1993), the increase in metabolism does not always imply an increase in neuronal activity. Thus, the dependence of systemic pressure and phrenic and peripheral nerve discharges on perfusion flow rate was investigated at room temperature.

**Figure 2:**
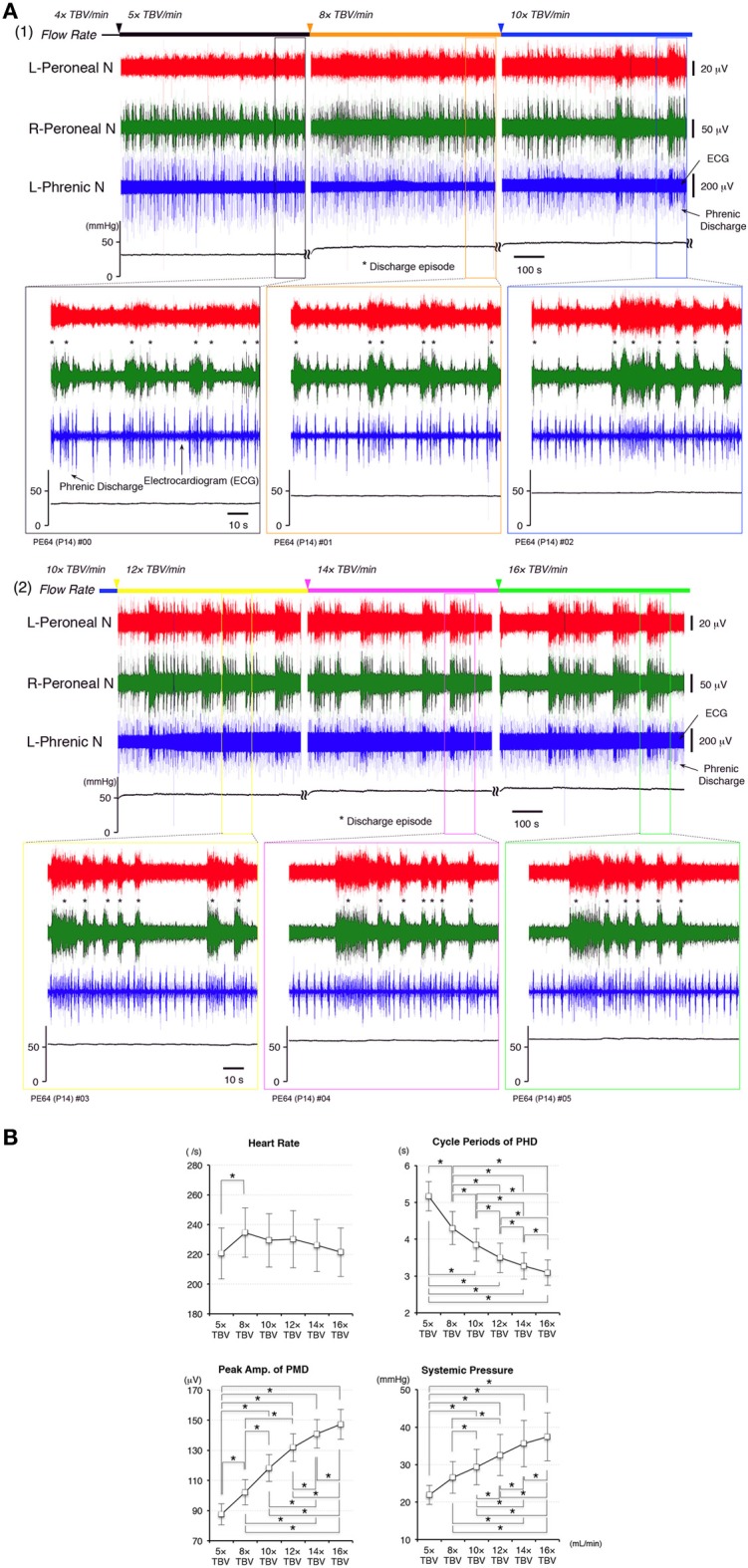
**(A)** Recordings showing perfusion flow dependence of systemic pressure and phrenic and peripheral nerve discharge in a decerebrate and arterially perfused *in situ* mouse preparation. **(1)** Data collected on perfusion flow dependence at 4× to 10× and **(2)** at 12× to 16× TBV/min. Phrenic discharge containing the ECG, and left and right peroneal nerve discharge were recorded at the various flow rates at room temperature. The upper panels show the systemic pressure, while the lower panels present an expanded view of the discharge and systemic pressure of the region surrounding that shown in the upper panels. All data were obtained from the same preparation made at postnatal day 14. As the flow rate increased to >10× TBV/min, the instances of phrenic and left and right peroneal nerve discharge became organized into discharge episodes (asterisks) of increasing incidence and duration punctuated by periods of quiescence. At the same time, the rhythm of the instances of left/right peroneal and phrenic nerve discharge during each discharge episode became more rapid, and small changes of systemic pressure occurred during discharge episodes. **(B)** Dependence of heart rate, systemic pressure, amplitude of the peripheral motor nerve discharge (PMD), and cycle periods produced outside of phrenic discharge episodes (PHD) on the flow rate tested. All data were obtained from the five preparations. The mean (SE) amplitude of the PMD and the systemic pressure increased with increased flow rate [*F*_(5, 4)_ = 23.696 and 14.363, *P* < 0.001] and significantly increased with each increase in flow rate (*P* < 0.05). The mean (SE) cycle periods of PHD outside of discharge episodes decreased with increased flow rate [*F*_(5, 4)_ = 79.069, *P* < 0.001] and significantly decreased with each increase in flow rate (*P* < 0.05). Although heart rate significantly increased at 8× TBV/min (*P* < 0.05), it decreased as the flow rate increased further [*F*_(5, 4)_ = 3.213, *P* < 0.05].

Figures 2A1,A2 shows examples of recordings from the left phrenic nerve, left and right peroneal nerves, and systemic pressure, in response to various perfusion flow rates (i.e., differing sympathetic tone). The ECG was obtained from the phrenic nerve recordings. All data were obtained from the same preparation. Both phrenic and peroneal nerve discharge amplitude and frequency increased with increases in flow rate. As the flow rate increased, the phrenic and bilateral peroneal nerve discharges became organized into “discharge episodes” of increasing frequency and duration, punctuated by periods of quiescence. The three discharge episodes were generated approximately at the same time. At flow rates of <10× TBV/min, the left and right peroneal nerve discharges during discharge episodes displayed “burst-like” activity. However, at flow rates of >10× TBV/min, they clearly showed rhythmic activity during discharge episodes. Although the rhythmic activity of the phrenic discharge increased with the increasing flow rates tested, it increased further when phrenic discharge episodes were generated (i.e., phrenic discharge episodes).

I investigated the dependence of heart rate, systemic pressure, amplitude of peripheral motor nerve discharges, and cycle periods of phrenic discharges outside of discharge episodes on perfusion flow rate. Recording periods of 10 min were set for each flow rate and data recorded during the last 5 min were used. All data were obtained from five preparations.

**Figure 3:**
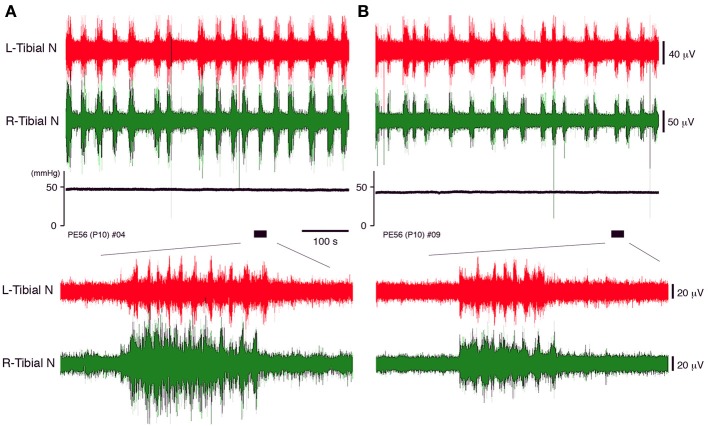
**The neuronal discharge and systemic pressure **(A)** before and **(B)** after bilateral vagotomy of a decerebrate and arterially perfused *in situ* preparation made at postnatal day 10 at the cervical level recorded at a flow rate of 11× TBV/min**. The lower panel provides an expanded view of the underlined portion of the neuronal discharge recording in the upper panel. The same results were obtained in all cases (*n* = 5).

In Figure 2B, the mean (SE) amplitudes of the peripheral motor nerve discharges and systemic pressure increased with increased flow rate [*F*_(5, 4)_ = 23.696 and 14.363, *P* < 0.001] and significantly increased with each increase in flow rate (*P* < 0.05). Also, the mean (SE) cycle periods of phrenic discharge outside of discharge episodes decreased with increased flow rate [*F*_(5, 4)_ = 79.069, *P* < 0.001] and significantly decreased with each increase in flow rate (*P* < 0.05). However, although heart rate significantly increased at 8× TBV/min (*P* < 0.05), it decreased as the flow rate increased further [*F*_(5, 4)_ = 3.213, *P* < 0.05]. Interestingly, several small systemic pressure changes were elicited during and after the discharge episodes in the phrenic nerve, including a eupnoeic pattern in the phrenic discharge at all of the flow rates tested. Similar results to those shown in Figure 3B were reproduced in all cases (*n* = 60).

### Effect of vagotomy on peripheral motor nerve discharges during discharge episodes

Vagotomy may contribute to the decrease in the peripheral nerve discharges during discharge episodes because it induces loss of sympathetic tone in the cardiovascular center of the brainstem and increases heart rate (Manning et al., 1963; Bromley and Holdstock, 1969). Although no neuronal discharges in the peripheral motor nerve were produced in the preparation unless the phrenic nerve discharge was generated (data not shown), the discharge episodes in which rhythmic activity was generated in the left phrenic and left and right peripheral motor nerves were produced by increasing the flow rate (Figure 3). But it is unclear whether they were affected by loss of sympathetic tone. Thus, the extent to which peripheral nerve discharges during discharge episodes were affected by vagotomy was investigated.

In decerebrate, arterially perfused mice, on postnatal day 10, the left and right tibial nerve discharges consisting of rhythmic activity during discharge episodes were compared before and after bilateral vagotomy at the cervical level (flow rate 11× TBV/min). The same pattern of neuronal activity during episodic discharges was seen before and after vagotomy (Figure 3). After vagotomy, the mean (SE) systemic pressure decreased by 7.71 (1.42)% (*P* < 0.05); the mean peak (SE) amplitude of the left and right tibial nerve discharge during discharge episodes decreased by 13.63 (2.34)% and 15.76 (2.71)% (*P* < 0.05), respectively; and the mean (SE) duration of the discharge episodes decreased from 11.91 (1.33) to 9.15 (1.75) s (*P* < 0.05), whereas the mean (SE) heart rate increased by 10.14 (2.5) % (*P* < 0.05). All data were obtained in each of the five preparations at the same flow rate (11× TBV/min). Recording periods of 10 min were set for each flow rate and data recorded during the last 5 min were used. These results indicate that the extent of left–right tibial nerve discharges during discharge episodes depends on the sympathetic tone in the brainstem.

### Phase relationships between left and right tibial nerve discharge during discharge episodes

**Figure 4:**
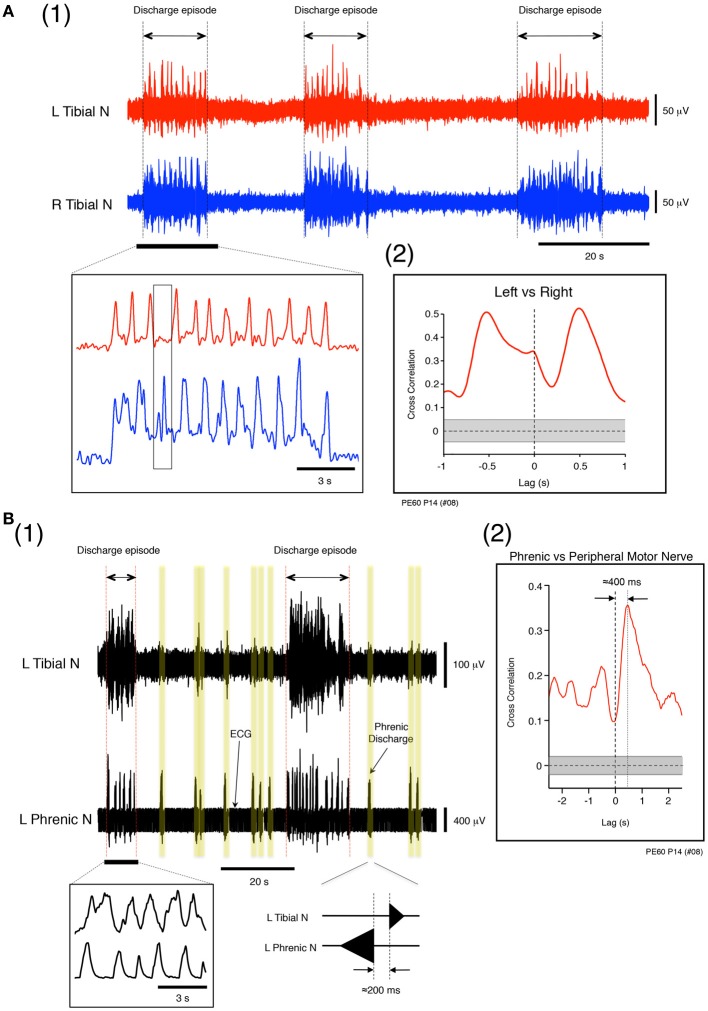
**(A) Left and right tibial nerve activity at a flow rate of 12× TBV/min. (1)** Recording showing the organization of neuronal activity into discharge episodes punctuated by quiescence. The results of rectification of the neuronal discharge episode appearing in the underlined part of the upper panel are shown in the left lower panel. The speed of the left and right alternating discharge was <2 Hz. **(2)** Recording showing correlation between instances of left and right tibial nerve discharge during discharge episodes. To determine the time lag between the two types of discharge during discharge episodes and the correlation in the time domain between lags from −1 to 1 s, time series data for the instances of left and right tibial nerve discharge during discharge episodes were obtained from the underlined data in the upper panel of **(1)**. In **(2)**, the left tibial nerve discharge positioned the right tibial nerve discharge to the right of vertical dotted line, and the right tibial nerve discharge positioned the left tibial nerve discharge to the left of the vertical dotted line. Each peak between the two gapped at ≈0.5 s (*r* = 0.5). The vertical dotted line denotes zero phase lags. The gray region represents the confidence bounds, which enclose only lags in samples that were not significantly correlated. **(B)** Left phrenic and left tibial nerve activity at a flow rate of 12× TBV/min. **(1)** Instances of neuronal discharge organized into discharge episodes during which phrenic nerve discharge was synchronous with tibial nerve discharge and punctuated by periods of quiescence. Between discharge episodes, the time lag between instances of phrenic and the tibial nerve discharge (yellow regions) was ≈200 ms. **(2)** Results of cross-correlation analysis of left phrenic and left tibial nerve discharge (peripheral motor nerve discharge) during discharge episodes to determine the time lag between the two types of discharges. The time series data for phrenic and tibial nerve discharge were obtained from all discharge episodes observed in **(1)** at this flow rate (12× TBV/min). One experiment was performed at the flow rate for 10 min, and most data for time-series episodes were extracted from the data collected from one experiment. Cross-correlation analysis of left phrenic and left tibial nerve discharge during discharge episodes and computation of the correlation in the time domain between lags from −2.5 to 2.5 s were also performed. In **(2)**, the phrenic discharge positioned the tibial discharge to right of the vertical dotted line, and the time lag between the two types of discharge within episodes was ≈400 ms (*r* = 0.35). The vertical dotted line denotes zero phase lags. The gray region represents the confidence bounds, which enclose only lags in samples that are not significantly correlated. Note that the results shown in **(A)** and **(B)** were obtained from the same preparation made at postnatal day 14.

The phase relationship between the left and right tibial nerve discharge during discharge episodes was investigated. At a flow rate of 12× TBV/min, the left and right tibial nerve discharges during discharge episodes assumed a clear left-and-right alternating pattern (Figure 4A1) resembling that induced by administration of serotonin, N-methyl-D,L-aspartate (NMDA), and dopamine to *in vitro* developing spinal-cord preparations (Whelan et al., 2000). Using the time series data of instances of left and right tibial nerve discharge (underlined data in Figure 4A1), cross-correlation analysis was used to determine the time lag from −1 to 1 s between the instances of left and right tibial nerve discharge. Each mean (SE) peak between instances of left and right tibial nerve discharge was characterized by a gap of 497.86 (22.82) ms (*r* = 0.5) (Figure 4A2). This result indicates that the frequency of left–right alternating discharge during discharge episodes was ≈1 Hz. The mean (SE) frequency of left–right alternating discharge episodes was 1.04 (0.01) Hz whenever left–right alternating discharge episodes were generated at the flow rates tested (>10× TBV/min; room temperature) in all cases (*n* = 60).

### Phase relationships between left phrenic and left tibial nerve discharge during discharge episodes

During perfusion at a high flow rate (>10× TBV/min; room temperature), the phase relationship between the left phrenic and left tibial nerve discharge during discharge episodes was investigated. Figures 4B1,B2 shows examples of left phrenic and left tibial nerve discharge patterns. In the absence of the discharge episodes, the mean (SE) time lag between peaks of phrenic and tibial nerve discharge was 201.27 (12.74) ms (Figure 4B1 yellow regions). Cross-correlation analysis was performed on the time series data of phrenic and tibial nerve discharge patterns during all discharge episodes to characterize the time lag from −2.5 to 2.5 s (Figure 4B1). As is shown in Figure 4B2, the mean (SE) time lag between the phrenic discharge and the tibial discharge (peripheral motor nerve discharge) in discharge episodes was 395.17 (19.10) ms (note that the peak is to the right of the vertical dotted line; *r* = 0.35). This indicates that the coupling of respiratory and locomotor rhythms occurred at about a 1:1 frequency ratio. Similar results to those shown in Figures 4A,B were reproduced in all cases, regardless of the age of the mouse and the experimental conditions (*n* = 60).

### Effect of high flow rates on sympathetic tone

Afferent inputs from peripheral and central chemoreceptors modulate sympathetic/parasympathetic tone in the brainstem (Loeschcke, 1982; O'Regan and Majcherczyk, 1982; Nattie, 1998; Ballantyne and Scheid, 2001). In the present study, the pH before and after systemic perfusion was maintained within the physiological range, at a mean (SE) of 7.35 (0.05), in all cases (*n* = 60). Thus, the effect of the impulse originating from the pH/PCO_2_ sensor was not examined (as it was assumed not to vary) and the response of the peripheral chemoreceptor to PO_2_ (O'Regan and Majcherczyk, 1982) was examined as flow rate was increased.

**Figure 5:**
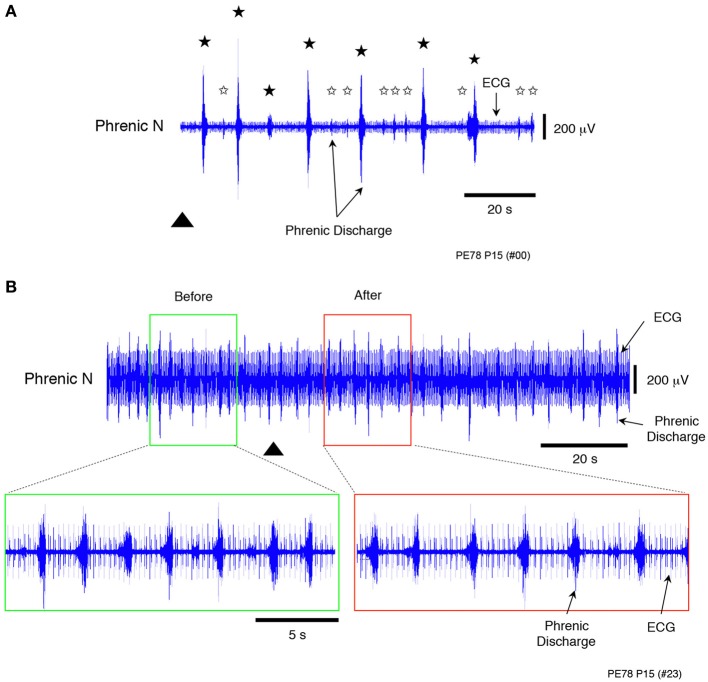
**Recordings showing the change in peripheral chemosensitivity with increasing flow rate when sodium cyanide (NaCN, a peripheral chemoreceptor stimulant), was applied to a decerebrated and arterially perfused preparation (500 μ M). (A)** Recording of chemoreflexes in the phrenic nerve discharge containing the ECG after NaCN (0.05 mL) had been applied at a low flow rate (6× TBV/min). **(B)** Recording of chemoreflexes in the phrenic nerve discharge containing the ECG after a large amount of NaCN (0.2 mL) had been applied at a high flow rate (16× TBV/min). Lower panels provide an expanded view of the phrenic discharge containing the ECG in the surrounding area appearing in the upper panels. In contrast to these results, no chemoreflexes had been induced in the phrenic nerve discharge before NaCN administration. Black stars **(A)** show chemoreflexes induced by NaCN, white stars the normal respiration pattern, and the triangle the time at which NaCN was applied. All results were obtained from the same preparation made at postnatal day 15. Similar results were seen in all cases (*n* = 4).

Sodium cyanide (NaCN; 500 μM), which is known as a chemoreceptor stimulant was applied to the decerebrate and arterially perfused preparations made at postnatal day 15. A chemoreflex and normal phrenic discharge were evoked in all cases (Figures 5A,B) when 0.05 mL NaCN was administered at a low flow rate (6× TBV/min). Conversely, a chemoreflex was never induced when a large amount of NaCN (0.2 mL) was administered at a high flow rate (16× TBV/min). The results in Figure 5 were obtained from five preparations.

These results and the decrease in heart rate seen at high flow rates (Figure 2B) indicate that loss of chemoresponse in the carotid body was probably due to saturation at high flow rates, as the amount of oxygen delivered to the preparation per unit of time increased with increasing flow rate. Thus, the modulated sympathetic tone induced by a hyperoxic/normocapnic state was found to activate the LPG via descending pathways to spontaneously produce a ‘locomotor-like’ discharge in the hindlimbs.

### Central mechanism(s) of the entrainment of respiratory and locomotor rhythms during discharge episodes

#### Origin of the discharge episodes in the decerebrate and perfused preparation

In general, respiratory rate decreases in a hyperoxic/normocapnic state at rest, but in the present preparation, respiratory rate increased even in a hyperoxic/normocapnic state (Figure 5B). Simultaneously, locomotor-like discharges were seen in the left and right tibial nerves during discharge episodes (Figure 4A). Furthermore, the time lag between phrenic and tibial nerve discharge during discharge episodes was ≈400 ms but not ≈200 ms (Figure 5B). These results led me to hypothesize that the mechanism(s) of increased respiratory rate during locomotion relies not only on input from the pH/PCO_2_ (Loeschcke, 1982; O'Regan and Majcherczyk, 1982; Nattie, 1998) and PO_2_ (Nattie, 1998) sensors (the central chemoreceptor and peripheral chemoreceptors, respectively) but also on other forms of input from the lower spinal cord. To test this hypothesis, a hindlimb preparation was developed to investigate whether the discharge episode obtained from the peripheral motor nerve (Figures 2, 4A) was produced by the lower spinal cord itself.

**Figure 6:**
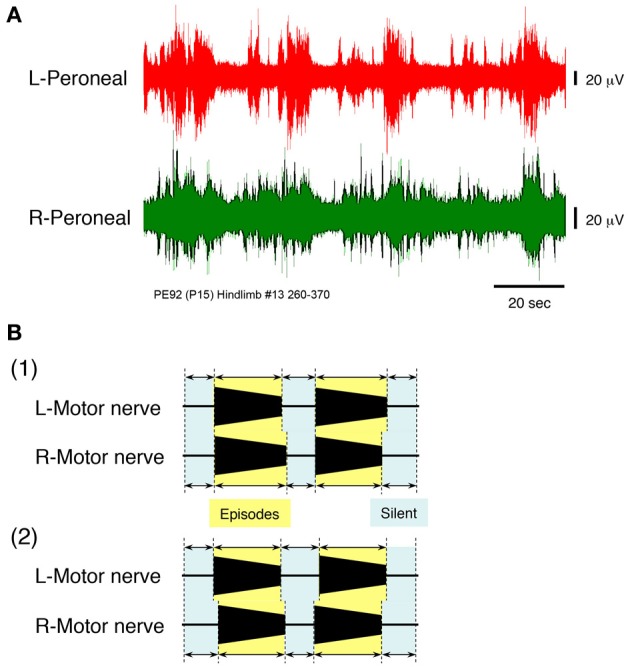
**(A)** Recordings showing the instances of left and right peripheral motor nerve discharge episodes induced by administration of high concentrations of serotonin, *N*-methyl-D,L-aspartate, and dopamine to a hindlimb preparation made at postnatal day 15. Neuronal discharge was recorded from the left and right peroneal nerves at a flow rate of 7× TBV/min. After administration of 160 μ M serotonin, 80 μ M *N*-methyl-D,L-aspartate, and 160 μ M dopamine, neuronal discharges became organized into episodes punctuated by periods of quiescence. The discharge episodes were induced simultaneously and periodically on both sides. Individual neuronal discharge episode consisted of a rhythmic and/or burst-like activity that occurred at various frequencies. The rhythmic discharge episode in the left and right peroneal nerve showed a left–right alternating pattern. The same result was obtained in all 12 hindlimb preparations. **(B1)** Schematic of the episodic pattern of discharge episodes, based on the discharge episode in Figures 3, 4A, produced at a high flow rate in a decerebrate and arterially perfused preparation. **(B2)** Schematic of the episodic pattern of discharge episodes, based on the discharge episode in **(A)**, induced by the administration of drugs to the hindlimb preparation. Periods in which discharge episodes were obtained in each nerve were defined as episodic periods and those in which no discharge episode was obtained were defined as silent periods. Both episodic and silent periods were recorded for each preparation and occurred simultaneously and repeatedly on both sides. Both episodic patterns were similar.

To induce the discharge episodes and/or burst-like discharge episodes shown in Figures 2, 4 in the lower spinal cord, high concentrations of serotonin, NMDA, and dopamine were applied to the hindlimb preparation at postnatal day 15 at a flow rate of 7× TBV/min, while neuronal discharge was recorded from the left and right peroneal nerves (Figure 6A). Administration of 160 μ M serotonin, 80 μM NMDA, and 160 μ M dopamine resulted in the generation of discharge episodes consisting of instances of rhythmic activity induced simultaneously and periodically on the left and the right sides, punctuated by periods of quiescence. With the rhythmic activity induced in the left and right peroneal nerve discharge showing a left–right alternating pattern. The same result was obtained at high concentrations of serotonin (60–160 μ M), NMDA (30–80 μ M), and dopamine (80–160 μ M) in all hindlimb preparations (*n* = 12).

Figure 6B1 shows a schematic of the episodic pattern, consisting of discharge episodes and no discharge episodes, based on the results shown in Figures 2, 4A (high flow rate, decerebrate, and arterially perfused preparation). Figure 6B2 is a schematic of the episodic pattern based on the results shown in Figure 6A induced by the administration of the drugs to the hindlimb preparation. The episodic periods (discharge episodes containing rhythmic and burst-like activity) and the silent periods (no discharge episodes) in each preparation were produced simultaneously and repeatedly on both sides. These results suggest that the discharge episodes in the left and right peripheral motor nerves originated in the lower spinal cord and not the brainstem.

#### The mechanisms of induction of the phrenic nerve discharge during discharge episodes

**Figure 7:**
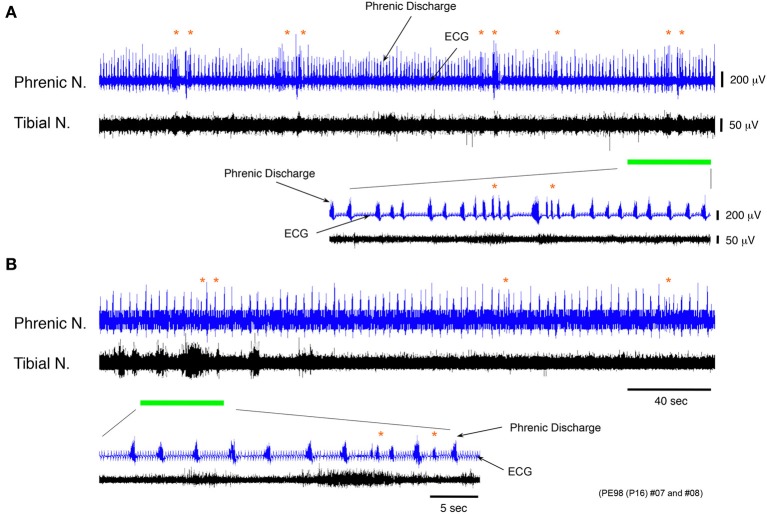
**Recordings of experimental trials showing changing incidence of phrenic discharge episodes before and after spinalization. (A,B)** Recordings showing phrenic and tibial nerve discharge induced before and after spinalization of a decerebrate and arterially perfused *in situ* preparation made at postnatal day 16 at the twelfth thoracic vertebra level at a flow rate of <×10 TBV/min. After spinalization, the frequency of phrenic discharge dramatically decreased and the mean (SE) incidence of phrenic discharge episodes per minute was 52.62 (11.96) % lower (*P* < 0.001). The respiratory rhythm after spinalization was regular compared with that before. The same result was obtained for all preparations (*n* = 3).

To confirm whether the phrenic nerve discharge episodes occurring during discharge episodes relies on impulses originating in the lower spinal cord, instances of phrenic and the tibial nerve discharge were recorded at flow rates of <10× TBV/min in a decerebrate and arterially perfused *in situ* preparation at postnatal day 16, spinalized at the twelfth thoracic vertebra to sever signal transmission to the brainstem from the lower spinal cord. When discharge episodes were periodically produced in the phrenic and tibial nerves, the same phase relationship between the phrenic and tibial nerve discharge, as that in Figure 4B, was seen in discharge episodes even after spinalization, but at the mean (SE) incidence per minute that was 52.62 (11.96) % lower (*P* < 0.001) (Figures 7A,B). In addition, the respiratory rhythm after spinalization was more regular than before. Although the episode discharge might be originated from the brainstem and the upper spinal cord, these results (obtained in each of three preparations), indicated that the locomotor-like discharge generated in the lower spinal cord produced a rapid phrenic discharge via ascending pathways and entrained the respiratory rhythm to the locomotor rhythm. They also suggest that a time lag of ≈400 ms is the time necessary for the impulse to travel from the lower spinal cord to the brainstem respiratory network via ascending pathways and then to the phrenic nerve via descending pathways.

## Discussion

**Figure 8:**
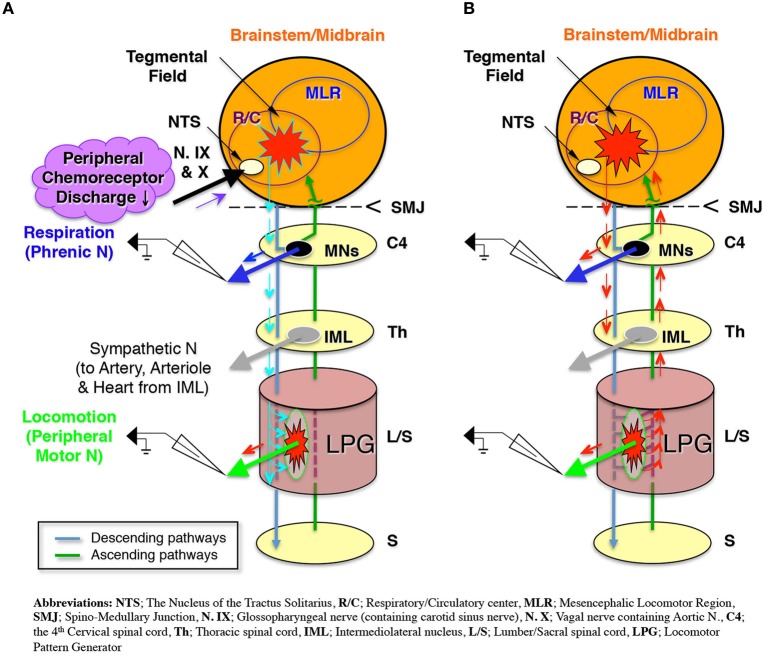
**Schematic representation of (A)**. The modulated sympathetic tone generating fictive locomotion via descending pathways and **(B)** The locomotor rhythm that entrains the respiratory rhythm via ascending pathways during locomotion.

The present study used extracellular recordings to investigate the neural pathways driving respiration and locomotion in a decerebrate and arterially perfused *in situ* mouse preparation. The results indicate that sympathetic tone modulated by a hyperoxic/normocapnic state activates the LPG through descending pathways and produces a locomotor-like discharge in the hindlimb (Figure 8A). After initiating this locomotor-like discharge, the generation of a locomotor-like discharge in the lower spinal cord acts on the brainstem respiratory center through ascending pathways to synchronize respiratory and locomotor rhythms (Figure 8B). These results indicate that (i) locomotion can be generated under specific respiratory conditions induced by hyperoxia/normocapnia and (ii) a spinal-feedback mechanism generating a locomotor-like discharge in the lower spinal cord is the principal mechanism in the entrainment of respiratory and locomotor rhythms. The existence of this mechanism implies: (i) that there is an autonomous reciprocal functional interaction between respiration and locomotion and (ii) that an increase in respiratory rate during locomotion is caused both by impulses from the lower spinal cord (which produces a locomotor-like discharge via ascending pathways), and afferent input received from mechanoreceptors and nociceptors.

### Sympathetic/parasympathetic tone of the decerebrate and arterially perfused *in situ* preparation

In the present study, the oxygen and ion components of body fluid required for the survival of the preparation were supplied by the perfusate via the blood vessels, while body temperature was maintained at room temperature. When the flow rate was high enough to produce a systemic pressure of >30 mmHg, the preparation showed spontaneous respiration, and when the perfusion flow rate was set at >5× TBV/min, the phrenic discharge showed a “eupneaic pattern” and “regular rhythm.”

The tip of the double-lumen catheter was held in the ascending aorta by the aortic valve and the internal pressure of the heart was maintained at atmospheric pressure. The lungs were completely removed. Thus, the effect of afferent input from mechanosensors of the heart wall (Bishop et al., 1983; Hainsworth, 1991; Hines et al., 1994), glomus type I cells on the carotid body that detect thermal changes (Alcayaga et al., 1993), and stretch receptors of the lung (Kalia and Sullivan, 1982; Hines et al., 1994) could be ignored. Because baroreflex-mediated parasympathetic/sympathetic control of vessel resistance is affected by pulsatile rather than non-pulsatile flow (James and de Burgh Daly, 1970; Chapleau et al., 1989), a peristaltic pump was used to provide a pressure pulse wave to the baroreceptors of the preparation, with pH before and after systemic perfusion continuously maintained within the physiological range. However, the preparation was gradually exposed to a hyperoxic/normocapnic state by increasing the flow rates (Figure 5). Simultaneously, the balance of the sympathetic/parasympathetic tone of the preparation broke down and the sympathetic tone was easily modulated. Thus, the decrease in heart rate shown in Figure 3B might have been induced because the sympathetic tone was modulated by the hyperoxic/normocapnic state. From the data obtained under these conditions, it appears that the sympathetic/parasympathetic tone of the preparation had been affected by the reception of afferent input from both the baroreceptors of the aortic bodies (James and de Burgh Daly, 1970; Chapleau et al., 1989), and the peripheral chemoreceptors of the carotid bodies (Nattie, 1998), although the sympathetic/parasympathetic tone increased with an increase in the flow rate. The results also indicate that the systemic pressure changes (Figure 2A) reflects not only changes in flow rate but also vasoconstriction and vasodilatation of both the arteries and the arterioles, which are controlled by sympathetic tone (Coleridge and Coleridge, 1980; Julius and Nesbitt, 1996).

In adult mice, the volume of blood circulated in a minute at rest is equal to the entire blood volume (Yang et al., 1999). Metabolism depends on both body temperature and perfusion flow rates: less oxygen is consumed at low body temperatures and high flow rates than at high body temperatures (Fox et al., 1982; Kirklin and Barratt-Boyes, 1993). Despite this, the 95% O_2_/5% CO_2_-infused perfusate used in the present study is clearly an inferior oxygen carrier compared with hemoglobin. Therefore, the experiments were performed at room temperature at a high flow rate to increase the amount of oxygen conveyed by the perfusate and minimize metabolism. When conducted in this manner, the systemic pressure of the preparation was much lower than that *in vivo*. This difference might be attributed to the following factors: (i) the deep hypothermia under which the preparation was examined (i.e., at room temperature), which led sympathetic tone to be extremely low; (ii) that despite increasing with increasing flow rate, the sympathetic tone seen at each high flow rate was easily modulated by afferent input from the peripheral chemoreceptors (Figure 2B), leading to a decrease in systemic blood pressure due to the hyperoxic state (Mokashi and Lahiri, 1991); (iii) the lack of viscoelasticity of the perfusate, compared with that of blood; and (iv) the leaking of perfusate from damaged capillaries and vessels when the preparation had been decerebrated and the skin systemically peeled. Furthermore, arginine vasopressin acts at V1 receptors to mediate vasoconstriction of peripheral blood vessels (Martin de Aguiler et al., 1990; Medina et al., 1998), changing the constrictor activity of the peripheral sympathetic nervous system (Bartelstone and Nasmyth, 1965). So the absence of arginine vasopressin from the perfusate, could also have played a role in the lower systematic respiration of the preparation compared with that *in vivo*.

The respiratory rhythm of the preparation was irregular not only compared with that *in vivo*, but also that of the *in situ* rat preparation of Pickering and Paton (2006). A hypothesis about the mechanism behind the induction of an irregular respiratory rhythm can be developed in accordance with the observation that the discharge episodes consisting of instances of left–right rhythmic or burst-like activity were generated in both the upper and the lower spinal cord at most of the flow rates tested. Consequently, the generation of the left–right alternating discharge in both the upper and the lower spinal cord affected the capability of the respiratory center to generate a regular respiratory rhythm, inducing an irregular respiratory rhythm.

## Conclusion

The findings of the present study indicate that specific respiratory conditions can generate locomotor-like discharge in the hindlimbs of mice. After a locomotor-like discharge is generated in the lower spinal cord, the LPG generating a locomotor-like discharge acts on the brainstem respiratory center through ascending pathways and thereby facilitates synchronization of the respiratory and locomotor rhythms. These results show that there are autonomous functions of respiration and locomotion. In addition, the increase in respiratory rate seen during locomotion is caused not only by reception of afferent input from mechanoreceptors and nociceptors but also from impulses from the lower spinal cord that produces a locomotor-like discharge via ascending pathways.

## Author contributions

Itaru Yazawa designed the study, analyzed the data, and wrote the paper.

### Conflict of interest statement

The author declares that the research was conducted in the absence of any commercial or financial relationships that could be construed as a potential conflict of interest.
